# Sex-Specific Changes in Gut Microbiome Composition following Blueberry Consumption in C57BL/6J Mice

**DOI:** 10.3390/nu11020313

**Published:** 2019-02-01

**Authors:** Umesh D. Wankhade, Ying Zhong, Oxana P. Lazarenko, Sree V. Chintapalli, Brian D. Piccolo, Jin-Ran Chen, Kartik Shankar

**Affiliations:** 1 Arkansas Children’s Nutrition Center, University of Arkansas for Medical Sciences, Little Rock, AR 72205, USA; SVChintapalli@uams.edu (S.V.C.); BDPiccolo@uams.edu (B.D.P.); ChenJinRan@uams.edu (J.-R.C.); ShankarKartik@uams.edu (K.S.); 2 Department of Pediatrics, University of Arkansas for Medical Sciences, Little Rock, AR 722025, USA; zhongying@uams.edu (Y.Z.); OPLazarenko@uams.edu (O.P.L.)

**Keywords:** microbiome, anthocyanins, blueberry, sexual dimorphism

## Abstract

The antioxidant and anti-inflammatory properties of blueberries improve vascular function and insulin sensitivity. However, the bioavailability of the active compounds in blueberries is largely dependent on the gut microbiota, which may themselves be altered by blueberry components. The objective of the current study was to explore a possible sex-dependent modulation of the gut microbiota following supplementation with blueberries in adult mice. Eight-week-old C57BL/6J mice (*n* = 7–10/group) were provided with control or blueberry-containing diets (5% freeze-dried powder) for 4 weeks. Body weight, composition, and food intake were measured weekly. Genomic DNA was isolated from the cecal contents for 16S rRNA sequencing. Blueberry feeding decreased α-diversity (operational taxonomical unit abundance) and altered β-diversity (*p* < 0.05). At the phylum level, the *Firmicutes* to *Bacteroidetes* ratio was significantly lower in the blueberry-fed groups (*p* < 0.001), along with increased *Tenericutes* and decreased *Deferribacteres*. At the genus level, blueberry feeding led to sexually-dimorphic differences, which were associated with predicted metabolic pathways. Pathways such as fatty acid and lipid metabolism were significantly different and demonstrated a stronger association with microbes in the male. To summarize, blueberry supplementation led to sexually-dimorphic global changes in the gut microbiome, which could possibly contribute to physiological changes in mice.

## 1. Introduction

Blueberries (*Vaccinium angustifolium*) have long been considered beneficial to human health. Nutritionally, one cup (100 g) of blueberries provides 3.6 g of fiber, 25% of the daily vitamin C requirement, and offers other nutritional benefits. Blueberry production has been on the rise in order to match the increasing level of consumption over the last several decades [1]. The health benefits of blueberries have been attributed to bioactive compounds such as ‘anthocyanins’ and ‘phenolic acids’ [2,3]. Anti-oxidant and anti-inflammatory effects of blueberries have been demonstrated in a range of studies using cellular systems and animal models [4,5,6]. Blueberry-derived anthocyanins have been reported to stimulate apoptosis and prevent the growth of cancer cells [7,8], as well as also having been shown to protect against oxidative stress [9]. In addition, dietary anthocyanins, phenolic acid, and blueberry supplementation were shown to improve hyperglycemia in diabetic mice, attenuate whole-body insulin resistance in high-fat diet (HFD)-fed mice, reduce inflammatory markers in adipose tissue, and aid in greater bone formation in rats [10,11]. 

Only 5%–10% of the biologically active compounds in blueberries are absorbed in the small intestine, while the remaining unabsorbed compounds reach the large intestine and colon, where they are available for microbial metabolism [12]. Many of these compounds are modified by gut microbes, resulting in polyphenol products which are known to mediate several physiological effects in the host [13]. Furthermore, the availability of anthocyanins in blueberries alters the composition of the gut microbiome by increasing the abundance of the microbes responsible for the metabolism and conversion of anthocyanins into bioactive xenometabolites [13], thereby demonstrating the impact of blueberry consumption on gut microbiota in rodents [14,15,16]. Considering that the digestibility and bioavailability of blueberries are contingent upon gut microbiota, studies to determine how blueberry consumption impacts gut microbial ecology are warranted. 

The influence of sex and diet on gut microbiota is well-established [17,18,19,20]. Changes in diet rapidly alter the composition of microbiota by changing the nutritional environment available for microbes within the gastrointestinal tract [17,21,22,23]. In contrast, sex has been theorized to alter the composition of gut microbiota via an interaction between microbes and sex hormones [18]. Although the exact mechanism underlying this interaction is not fully understood, it appears to be elicited by a sexually-dimorphic immune response [18,19,24,25]. However, most dietary interventions focusing on the gut microbiome do not account for the role of sex. Thus, the interaction between diet and sex, and its effect on the gut microbiome, remain to be fully explored in the context of blueberry consumption. 

In the current study, we investigated the effects of blueberry supplementation on the gut microbiota composition in male and female C57BL/6J mice. In addition, we determined whether an altered microbiome was associated with changes in the predicted metabolic functional pathways. Finally, we investigated sexually-dimorphic changes in the microbiome and metabolic function in male and female mice in response to the blueberry diet. Our study provides evidence of marked changes in the composition and functional potential of the gut microbiome following blueberry supplementation in mice. Importantly, these findings strongly suggest that blueberry feeding elicits these differences in a sexually-dimorphic manner. Furthermore, correlations among metabolically relevant functional pathways and microbial families demonstrate a stronger association in male mice relative to female mice. 

## 2. Materials and Methods

### 2.1. Experimental Design 

The mice were group-housed (4–5 mice per cage) in an Association for the Assessment and Accreditation of Laboratory Animal Care-approved animal facility at the Arkansas Children’s Research Institute, with a constant temperature of 22 °C and the lights on from 6:00 a.m. to 6:00 p.m daily. The Institutional Animal Care and Use Committee at the University of Arkansas for Medical Sciences approved all of the animal procedures. Four-week-old male and female C57BL/6J mice were produced from several breeding pairs using multiple dams. The male and female mice were randomly divided into four different groups. The number of mice per group was dictated by power analysis. Using the effect sizes (F_BB *n* = 9 and F_Con *n* = 7 for females; and M_BB *n* = 9 and M_Con *n* = 10 for males), we computed that we had over 76% power in males and over 93% power in females to detect differences at *α* = 0.05 between the blueberry diet groups with the samples sizes present herein. Power calculations were designed for the main outcomes, such as weight and body composition, but not for microbiome analysis since the anticipated effect sizes for each taxonomic unit differed and it was hard to estimate a priori. 

The mice were randomized to either a control diet (TD.95092, Harlan Teklad, MA, USA) or a blueberry-supplemented diet (containing 5% (by wt) freeze-dried blueberry powder (TD.10679, Harlan Teklad, MA, USA)) provided *ad libitum*. Freeze-dried whole blueberry (*Vaccinium angustifolium*) powder (Hi-Actives Wild Blueberry) was kindly provided by VDF/FutureCeuticals, Momence, IL, USA. The nutrient composition of both diets is provided in Table 1. Both diets contained casein as the sole protein source. Mice were divided into four groups: Male mice fed a control diet (M_Con, *n* = 10), male mice fed a blueberry diet (M_BB, *n* = 9), female mice fed a control diet (F_Con, *n* = 7), and female mice fed a blueberry diet (F_BB, *n* = 9). During the 28-day dietary intervention, body weights were measured weekly and body composition was measured by Echo MRI at the end of the study (at 8 weeks). The mice were euthanized at ~9.00 a.m. in the fed state by carbon dioxide asphyxiation and the cecal contents and other tissues (adipose tissue, liver, muscle, and bone) were collected and snap-frozen for further analysis.

### 2.2. Microbial Community Profiling Using 16S rRNA Amplicon Sequencing

Genomic DNA was extracted from the cecal contents using the MO BIO PowerSoil DNA Isolation kit (Qiagen, Gaithersburg, MD, USA) with a few modifications. The cecal contents (20–25 mg) were added directly into 96-well plates with beads and recommended buffers in the wells. The sealed plates were shaken horizontally at 20 rpm for 20 min using the MO BIO shaker. The remaining steps were performed as directed by the manufacturer. The extracted DNA was quantitated spectrophotometrically and stored at −20 °C. Fifty nanograms of genomic DNA were utilized for the amplification of the V4 variable region of the 16S rRNA gene using 515F/806R primers. Forward and reverse primers were dual-indexed, as described by Kozich et al., to accommodate the multiplexing of up to 384 samples per run [26]. Paired-end sequencing (2 × 250 bp) of pooled amplicons was carried out on an Illumina MiSeq with ~30% of PhiX DNA [27]. 

### 2.3. Bioinformatics Analysis

The processing and quality-filtering of reads were performed by using scripts in QIIME (v1.9.1) [28] and other in-house scripts [29]. Paired reads were stitched with PEAR, an over-lapping paired-end reads merger algorithm, which evaluated all possible paired-end read overlaps, minimizing false-positive hits [30]. The reads were further filtered based on Phred quality scores and, for chimeric reads, using USEARCH61 [31]. The filtered reads (mean counts per sample = 37,971) were de-multiplexed within QIIME and samples with less than 5000 reads were excluded from further analysis. UCLUST was used to cluster sequences into operational taxonomical units (OTUs based on >97% identity) [31]. Operational taxonomical unit (OTU)-picking was performed using an open-reference method which encompassed the clustering of reads against a reference sequence collection, as well as picking out the reads which failed to align with any known reference sequence in the database [32]. To eliminate erroneous mislabeling, the resulting OTU tables were checked for mislabeling sequences [33]. Representative sequences were further aligned using PyNAST with the Greengenes core-set alignment template [34]. Construction of a phylogenetic tree was performed using the default (FASTTREE) method in QIIME [35]. OTUs were normalized by the predicted 16S copy number and functions were predicted with the use of GreenGenes 13_5 databases for KEGG Orthologs.

PICRUSt, a bioinformatics software package designed to predict metagenome functional content from marker gene surveys (e.g., 16S rRNA) and full genomes, was used to identify differences in predictive metagenome function [36]. 

### 2.4. Statistical Analysis

The microbiota OTU reads were imported into the R version 3.4.3 (https://cran.r-project.org/) and all statistical analyses were performed using the vegan and phyloseq packages unless specifically noted. The OTU richness was measured by Chao1, while the OTU diversity was measured by several diversity indices (Shannon, Simpson, Inverse Simpson, and Fisher). Group differences in α-diversity (richness and diversity) were assessed by ANOVA. Between-specimen diversity (β-diversity) was assessed by using Bray−Curtis dissimilarities and was then visualized using non-metric multi-dimensional scaling (NMDS). The treatment group differences in β-diversity were assessed on the dissimilarity matrix using permutational multivariate analysis of the variance (PERMANOVA) with 500 permutations. Group differences among taxa-level OTUs were assessed by pairwise comparisons between read counts using Negative Binomial Wald Tests from the DESeq2 package. The OTU relative abundance is given as the median % relative abundance when described in the text. All of the statistical tests used on 16S-rRNA gene sequencing data were considered significant, at *p*  ≤  0.05. All of the tests were corrected for multiple comparisons using the false discovery rate (FDR) correction by Benjamini and Hochberg. Associations among selected variables were assessed with Spearman’s correlations. An in-house developed R-based Shiny app (DAME) was utilized to facilitate the procedures and statistical analysis described above [37]. All of the statistical analyses were performed and figures were made using R (version 3.4.3, https://cran.r-project.org/). Two-Way ANOVA was used to determine the main effects of the blueberry diet and sex separately, as well as their interactions. The correlation between microbial abundance and predicted metagenomic function was performed using the corrplot package in R and the utilized microbe abundance at the family or genus levels, as described in the specific comparison. The statistical significance was determined at *p* ≤ 0.05. 

## 3. Results

### 3.1. Body Weight, Body Composition, and Tissue Weights 

**Figure 1:**
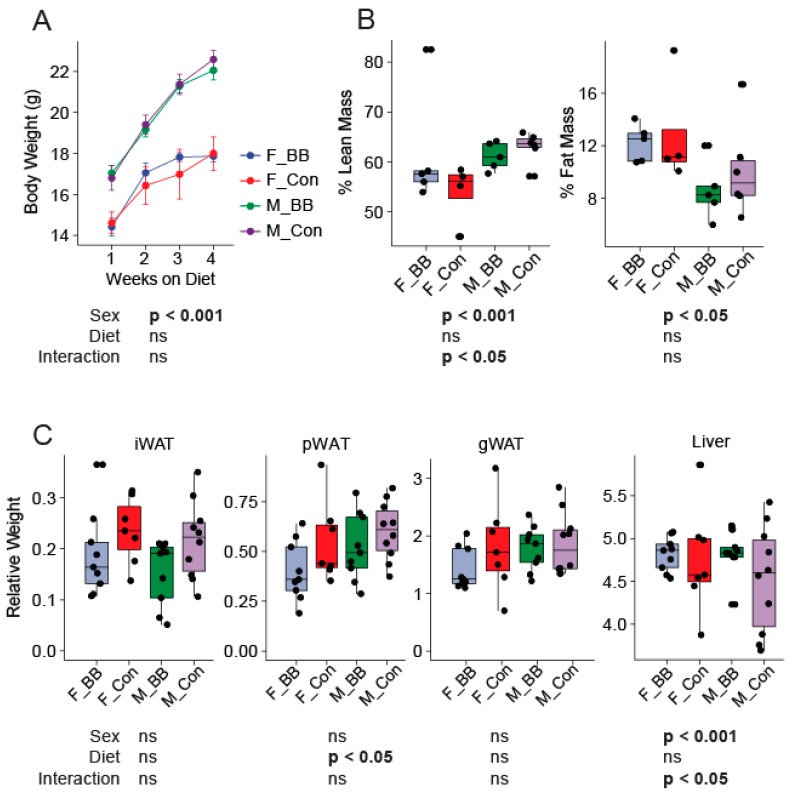
Body weight, body composition, and tissue weights of mice during the dietary intervention. (**A**) The body weight of male and female mice over the four weeks of dietary supplementation, (**B**) The body composition (% Lean mass and % Fat mass) at the end of the dietary intervention, and (**C**) iWAT (inguinal), pWAT (perirenal), gWAT (gonadal) white adipose tissue weights expressed relative to body weight at the time of euthanization. F-BB (female mice fed a blueberry diet) (*n* = 9), F-Con (female mice fed a control diet) (*n* = 7), M-BB (male mice fed a blueberry diet) (*n* = 9), and M-Con (male mice fed a control diet) (*n* = 10). A Two-Way ANOVA analysis was performed using R. Sex and diet and the interactions of both were used as two factors to determine the significance. (ns = non-significant).

Dietary blueberry supplementation did not alter the body weights in male and female mice between the blueberry and control diet-fed groups over the four week study period (Figure 1A). The food intake was not different amongst groups (data not shown). There were no diet-associated differences in the percent of fat mass or the percent of lean mass, as measured by MRI, at the end of the study between the groups fed the control or the blueberry diet (Figure 1B). However, the well-documented sex differences in weight gain and body composition were evident. Male mice weighed significantly more than female mice over the four week period (Sex, *p* < 0.001). The percent of lean mass was greater in male mice relative to female mice and the percent of fat mass was reduced in male mice compared to females (Sex, *p* < 0.05) (Figure 1B). The relative weights of the inguinal (iWAT) fat depot, gonadal (gWAT) fat depot, and liver normalized to body weight were not different, except for perirenal fat depots (pWAT), which were significantly higher (*p* < 0.05) in the groups fed the blueberry diet (Figure 1C). 

### 3.2. Gut Microbial Taxonomic Analysis

**Figure 2:**
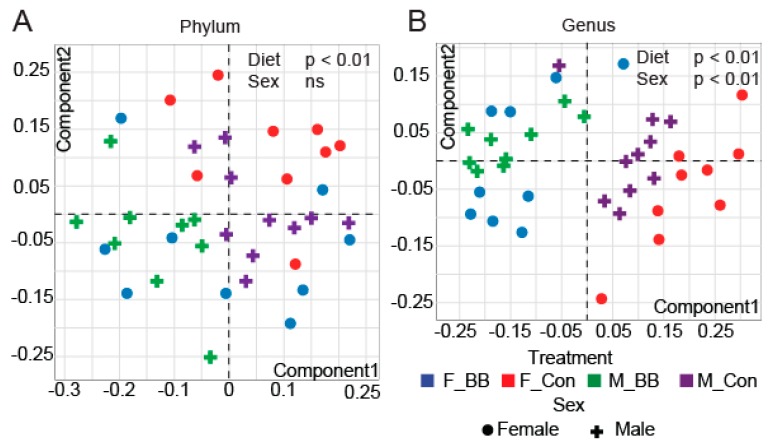
The β-diversity of microbial ecology upon blueberry supplementation. Non-metric multi-dimensional scaling (NMDS) analysis of the operational taxonomical unit (OTU) abundance matrix of the β-diversity of gut microbial communities shows differences at both the (**A**) phylum and (**B**) genus level. (F-BB (female mice fed a blueberry diet) (*n* = 9), F-Con (female mice fed a control diet) (*n* = 7), M-BB (male mice fed a blueberry diet) (*n* = 9), and M-Con (male mice fed a control diet) (*n* = 10). Multi-factor ANOVA analysis was performed using an R-based Shiny app (DAME). Sex and diet were used as two factors to determine the significance (ns = non-significant).

We analyzed the gut microbiota composition by 16S rRNA amplicon sequencing of cecal contents and observed distinct differences in the microbial taxa associated with blueberry consumption. Microbiome diversity is typically described in terms of within (i.e., α) and between sample (i.e., β) diversities. α-diversity indices, such as Chao1 and the number of Observed OTUs (measures of richness), were significantly increased in blueberry-fed groups in males and reduced in females at the phylum level (Diet × Sex, *p* < 0.05) (Table 2). At the genus level, blueberries significantly reduced the total observed OTUs and the Chao1 and Fisher indices compared to the control groups in both male and female blueberry-fed mice (Diet, *p* < 0.05) (Table 2). Non-metric multi-dimensional scaling (NMDS) ordination plots of Bray−Curtis dissimilarities revealed significant differences in β-diversity at all taxonomic levels due to blueberry supplementation in both male and female mice (Diet, *p* < 0.01). However, sex affected β-diversity at the genus level (*p* < 0.01) but not at the phylum level (Figure 2A,B). 

### 3.3. Blueberry Consumption-Associated Taxonomical Differences

**Figure 3:**
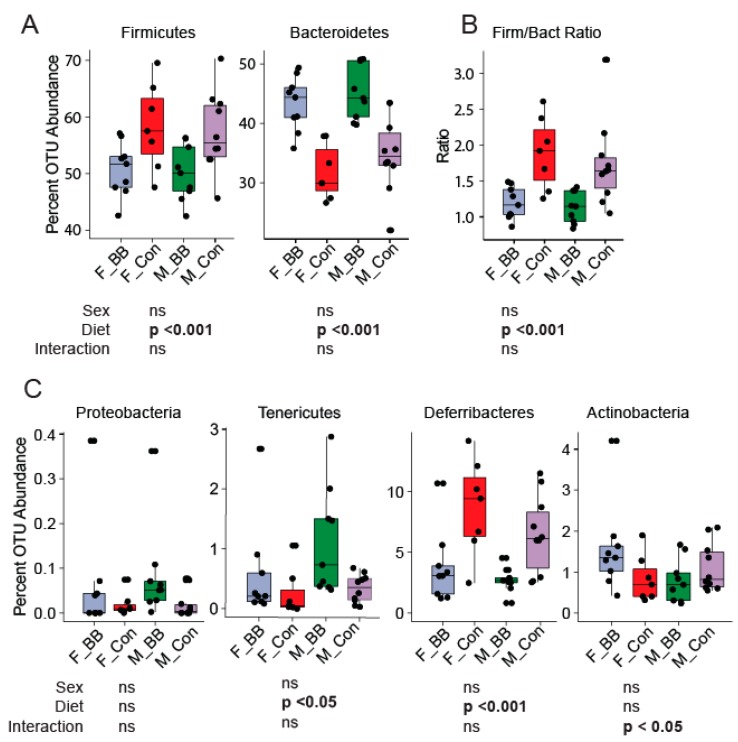
Operational taxonomical unit (OTU) abundance at the phylum level upon blueberry supplementation. (**A**) The OTU abundance of *Firmicutes* and *Bacteroidetes* was decreased and increased, respectively, in the blueberry-supplemented groups in both sexes. (**B**) Decreased *Firmicutes* to *Bacteroidetes* ratios in the blueberry groups were seen. (**C**) *Proteobacteria, Tenericutes, Deferribacteres,* and *Actinobacteria* were also altered by dietary blueberry supplementation. F-BB (female mice fed a blueberry diet) (*n* = 9), F-Con (female mice fed a control diet) (*n* = 7), M-BB (male mice fed a blueberry diet) (*n* = 9), and M-Con (male mice fed a control diet) (*n* = 9). Two-Way ANOVA analysis was performed using R. Sex, diet, and their interaction, then these two factors were used to determine the significance. (ns = non significant).

Gut microbiome assessment of specific bacterial phyla revealed significant differences due to supplementation in both male and female mice. *Bacteroidetes’* abundance increased, whereas *Firmicutes’* abundance decreased significantly in mice fed the blueberry diet (Diet, *p* < 0.001) (Figure 3A). The *Firmicutes* to *Bacteroidetes* ratio was lower in both male and female mice fed blueberry diets compared to mice fed a control diet (*p* < 0.001) (Figure 3B). *Deferribacteres* was lower in blueberry-fed mice compared to control diet-fed mice (*p* < 0.05) (Figure 3C). *Tenericutes* was higher in blueberry-fed mice in both males and females (*p* < 0.05) (Figure 3C). 

**Figure 4:**
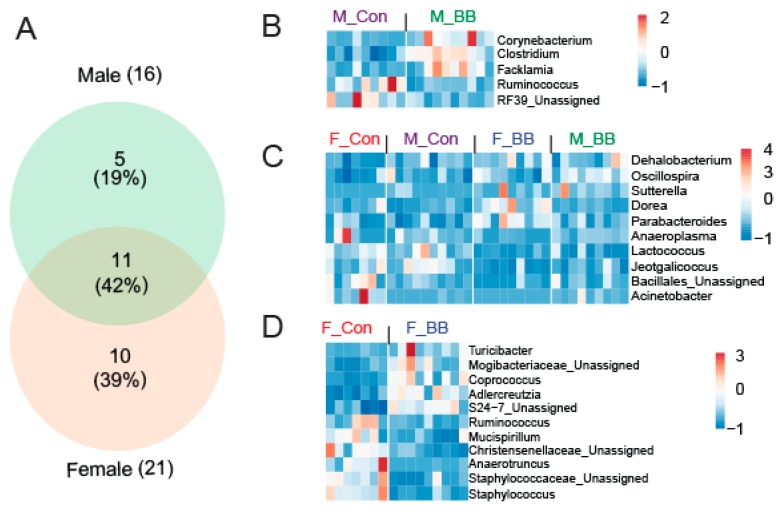
Blueberry supplementation affects the differential abundance of genera in a sexually-dimorphic pattern. (**A**) A Venn diagram showing the differential abundance of unique and common genera when determining the effect of blueberry supplementation in male and female mice. A heat map of uniquely expressed and significant genera in (**B**) males only, (**C**) males and females, and (**D**) in females only. F-BB (female mice fed a blueberry diet) (*n* = 9), F-Con (female mice fed a control diet) (*n* = 7), M-BB (male mice fed a blueberry diet) (*n* = 9), and M-Con (male mice fed a control diet) (*n* = 9). The color legend represents the relative fold (normalized) abundance of particular genera. The color scale shows the fold change. (Differential abundance analysis was performed using Negative Binomial Regression by taxonomic levels with pairwise comparisons of meta-data using DESeq2 workflow).

At the genus level, a significant effect of blueberry-feeding was present in 16 genera in males and 21 genera in female mice (*p* < 0.05). When assessed for commonality and uniqueness among the genera, five genera were unique in males and 10 genera were unique in females, whereas 11 genera were commonly affected in both male and female mice (Figure 4A) following blueberry consumption. Of the five genera unique to males, *Corynebacterium, Clostridium,* and *Facklamia* were increased while *Ruminococcus* and *RF39* were decreased in the blueberry-fed groups (Figure 4B). In female mice, *Turicibacter, Mogibacteriaceae, Coprococcus, Adlercreutzia,* and *S24-7* were increased while *Ruminococcus, *Mucispirillum***,** Christensenellaceae, Anaerotruncus,* and *Staphylococcus* were decreased in the blueberry-fed groups (Figure 4D). Genera affected by the blueberry diet across both sexes included *Dehalobacterium, Oscillospira, Sutterella, Dorea, Parabacteroides, Anaeroplasma, Lactococcus, Jeotgalicoccus, Bacillus,* and *Acinetobacter* (Figure 4C). The detailed relative percent of the OTU abundance at the genus level for individual genera can be found in Table 3. 

### 3.4. PICRUSt-Predicted Metabolic Pathways 

**Figure 5:**
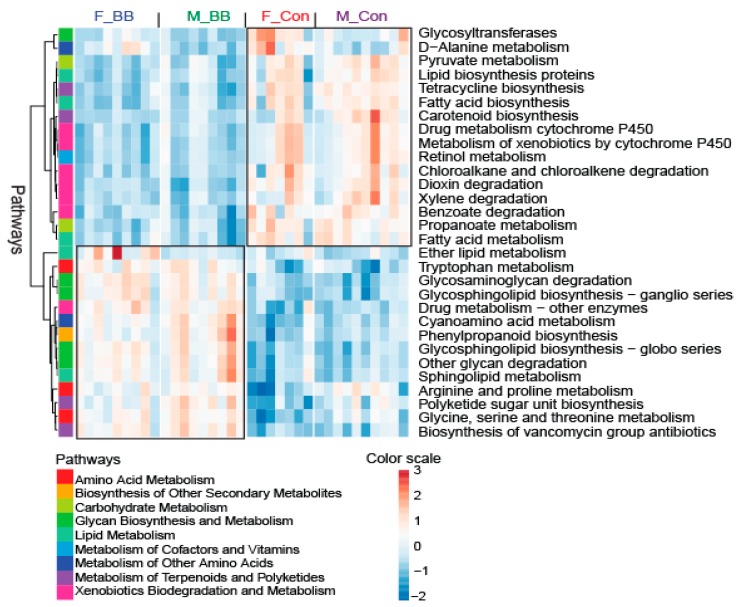
The PICRUSt-predicted biological function pathways upon blueberry supplementation. The metabolic pathways from KEGG module predictions used 16S data with PICRUSt and sequenced shotgun metagenomes. F-BB (female mice fed a blueberry diet) (*n* = 9), F-Con (female mice fed a control diet) (*n* = 7), M-BB (male mice fed a blueberry diet) (*n* = 9), and M-Con (male mice fed a control diet) (*n* = 9).

Overall, blueberry-feeding had a major effect on the predicted metabolic pathways in both male and female mice, based on PICRUSt analysis. Metabolism-related pathways, such as pyruvate metabolism, fatty acid biosynthesis and metabolism, metabolism of xenobiotics, and retinol metabolism, were less-represented in the collective microbial genome in blueberry-fed mice compared to control-fed mice (Figure 5). The abundance of genome encoding factors related to lipid metabolism, tryptophan metabolism, arginine and proline metabolism, and polyketide and sugar unit biosynthesis were higher in control diet-fed mice as compared to blueberry-fed mice (Figure 5). 

**Figure 6:**
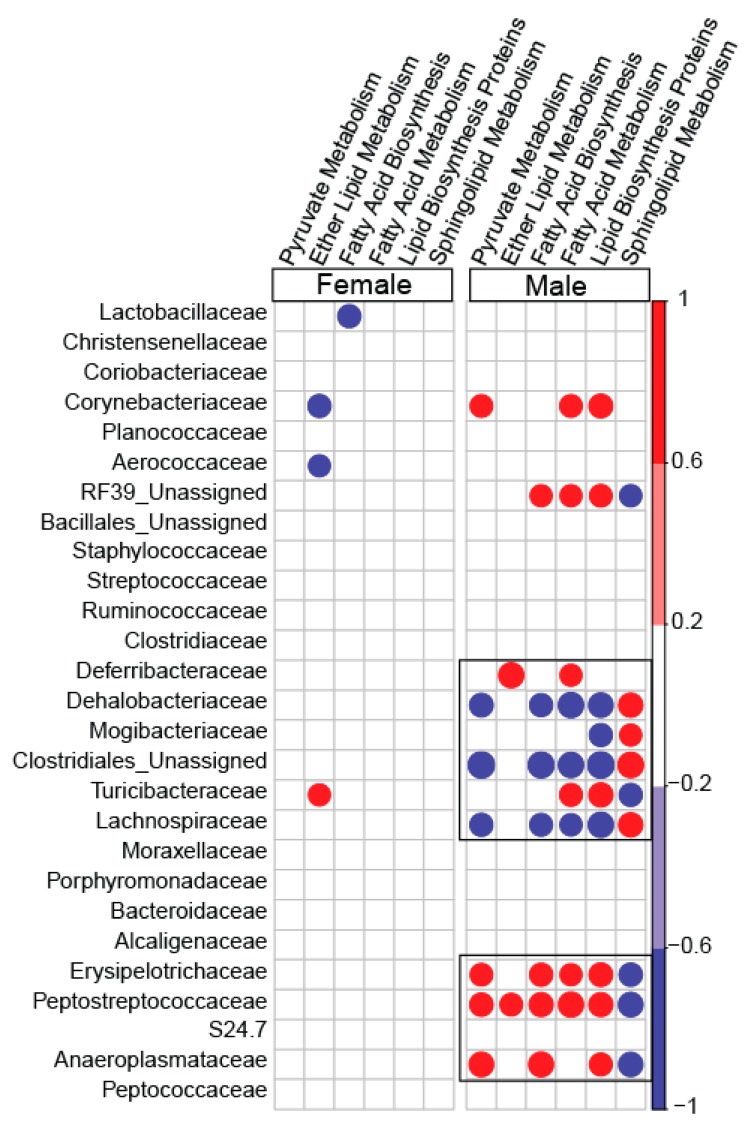
Spearman correlations of specific metabolic pathways predicted from microbial genomes, with the operational taxonomical unit (OTU) abundance of families. Pathways such as lipid metabolism, fatty acid metabolism, lipid biosynthesis, etc. and their association with OTU abundances at family-level taxa are shown. The color red denotes a positive association, whereas the color blue denotes negative correlations; only statistically significant correlations are depicted. The color gradient represents the degree of association.

**Figure 7:**
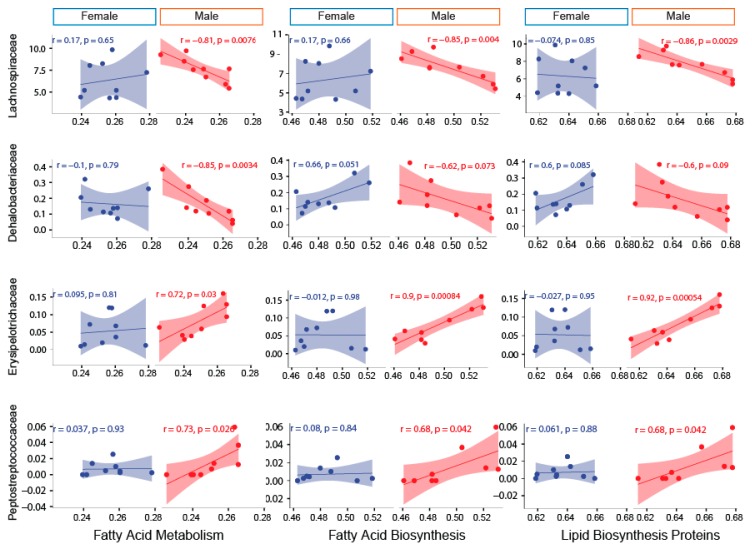
The sexually-dimorphic association of fatty acid metabolism, fatty acid synthesis, and lipid biosynthesis with Lachnospiraceae, Dehalobacteriaceae, Erysipelotrichaceae, and Peptostreptococcaceae families. PICRUSt-generated predicted metabolic pathways (the mean relative frequency % based on operational taxonomical unit (OTU) abundance) such as fatty acid metabolism, fatty acid biosynthesis, and lipid biosynthesis show a sexually-dimorphic association with Lachnospiraceae, Dehalobacteriaceae, Erysipelotrichaceae, and Peptostreptococcaceae

To determine the association between the family-level abundance and predicted metabolic pathways, five important pathways under the metabolism category were picked. The sexually-dimorphic differences of these pathways and their overall significance in metabolism were the basis for final selection. Pyruvate metabolism, ether lipid metabolism, fatty acid biosynthesis, fatty acid metabolism, lipid biosynthesis protein, and sphingolipid metabolism demonstrated a stronger association with microbial families in blueberry-fed male mice compared to blueberry-fed female mice. Lachnospiraceae and Dehalobacteriaceae were negatively correlated with fatty acid metabolism, including lipid biosynthesis, in male blueberry-fed mice. Erysipelotrichaceae and Peptostreptococcaceae were positively associated with fatty acid metabolism, including lipid biosynthesis, in only male mice. There were no correlations between the aforementioned families and the metabolic pathways in female mice (Figure 6 and Figure 7). 

## 4. Discussion

To our knowledge, there are no reports documenting sexually-dimorphic differences in gut microbiota in response to blueberry supplementation. In the current study, the central question addressed is three-fold: Whether blueberry dietary supplementation led to altered gut microbiota, whether these changes were distinct in male and female mice, and whether the altered microbiome composition was associated with differences in the predictive metabolic function. Remarkably, blueberry diets not only altered the overall microbiome composition but also did so in a sexually-dimorphic manner. These changes translated to changes in the predicted metabolic functional pathway as well. Sexual dimorphism persisted in changes in the predictive metabolic functional pathways, i.e., male mice demonstrated stronger associations with fatty acid and lipid-related pathways compared to their female counterparts on a blueberry diet. 

Along with the well-defined impact of sex on physiology and behavior [38], sex-based differences in the gut microbial composition have also been increasingly documented [39,40,41]. Recent studies have provided evidence for differences in gut microbiota composition between the sexes and for the specific influence of diet, as well as sex hormones, on gut microbiota composition [18,42,43]. Factors including allosomally-linked genes, a differential abundance of sex hormones, differential body composition, altered cytokine and hormone profiles, and a distinctive gut microbiota have been implicated in contributing to sexual dimorphism [43,44,45,46]. Of these factors, the role of gonadal steroids, especially estrogens and downstream signaling via estrogen receptors, have been extensively examined to explain sex differences in the metabolism [47,48]. We observed that a number of significantly affected microbial genera, in response to blueberry supplementation, were greater in female (21) compared to male (16) mice. The predicted biological functions of the observed microbial community in the control and blueberry-fed mice were significantly different to animals fed the control diet and followed the sexually-dimorphic pattern. Interestingly, we observed a stronger association among predicted meta-genomic pathways related to lipid and fatty acid metabolism and the Erysipelotrichaceae and Peptostreptococcaceae families in male mice, suggesting that blueberries may promote sex-dependent differences. However, the mechanisms contributing to the observed diet-sex interaction, in response to blueberry supplementation, remain to be studied using a more mechanistic approach. 

Blueberries are high in anthocyanins, in addition to other polyphenolic compounds [3], and have anti-oxidant and anti-inflammatory properties [49,50,51,52] which contribute to improved vascular function and increased insulin sensitivity [53,54]. Anthocyanins are not digested or metabolized in the stomach or upper intestine and remain intact until reaching the large intestine, where they are converted to phenolic acids by commensal bacteria [12,55]. It is likely that anthocyanin consumption alters the composition and function of the gut microbiota, leading to an enrichment of bacteria that enhances the metabolism of anthocyanin; however, this bidirectional relationship has not yet been addressed in anthocyanin research, to our knowledge. The current report contributes new knowledge to the repertoire of changes in microbiota associated with the consumption of this dietary factor.

Findings of the current study, showing altered microbial ecology in blueberry-fed mice, are in line with previous reports of other polyphenol-rich foods. In one study, a reduction in the ratio of *Firmicutes* to *Bacteroidetes* and an increase in *Akkermansia muciniphila* was observed following the intake of grape extract (1% Concord grape polyphenols) for 13 weeks in mice [56,57]. These changes conferred protection against the negative consequences of the high-fat diet (HFD), as shown by a reduction in systemic inflammation and an improvement in insulin sensitivity [56,57]. In the current study, OTU abundances of major phyla such as *Bacteroidetes* and *Tenericutes* were significantly increased in blueberry-fed mice, which is consistent with a recent study by Lee et al. where they fed blueberry powder (10% by weight) to rats [58]. Akin to our findings, the *Firmicutes* to *Bacteroidetes* ratio was significantly lower in blueberry-fed rats. There is evidence supporting the notion of *Firmicutes* as energy harvesters and *Bacteroidetes* as energy consumers, and their ratio might be indicative of the state of adiposity [59]. However, in the current study, the shift in the *Firmicutes: Bacteroidetes* ratio was not accompanied by any differences in adiposity, body weight, or other metabolic endpoints upon blueberry feeding. Liu et al. previously reported that mice fed grape seed proanthocyanidin extract had a higher abundance of Erysipelotrichaceae, Lachnospiraceae, and Peptostreptococcaceae families, which was associated with reduced inflammation and improved insulin sensitivity in mice [60]. Interestingly, we observed a positive association among the predicted meta-genomic pathways related to lipid and fatty acid metabolism and Erysipelotrichaceae and Peptostreptococcaceae families in blueberry-fed mice. This correlation was only observed in male mice, further supporting the idea that blueberry components may promote sex-dependent differences. It remains to be determined in human studies if similar interactions between anthocyanins, or other blueberry components, and the gut microbiota are present.

Blueberry-supplemented diets are known to improve glucose homeostasis and reduce body weight in experimental animals [53,61,62,63]. In our study, blueberry supplementation did not change the body weight or body composition in C57BL/6 mice. Several factors, including the relatively young age of mice (4 week old weanling mice), the shorter duration of dietary intervention (4 weeks), the feeding of blueberry powder rather than pure anthocyanins, and the absence of an obesogenic challenge (viz. high-fat diets) are all likely to have led to the results that we observed in our experiment. Consistent with our study, blueberry supplementation for shorter durations did not change the body weight or tissue weights in diabetic mice [64] and rats [58]. Nonetheless, the duration of dietary exposure was sufficient to dramatically reconfigure the gut microbiome of the mice, consistent with a predominant role of the diet on gut microbiota.

The predicted functional profiling of microbial communities provides an insight into the genetic make-up of the microbial population and how it might impact the host’s biological pathways [36]. The predicted biological functions of the observed microbial community in control and blueberry-fed mice were significantly different to those of control-fed animals. Blueberry supplementation during the current study impacted a total of 27 metabolic pathways irrespective of sex. Microbial pathways impacting pyruvate metabolism, lipid biosynthesis, fatty acid biosynthesis, metabolism of xenobiotics, and chloroalkane and chloroalkene degradation were lower in blueberry-fed mice. Pathways including tryptophan metabolism, cyanoamino acid metabolism, arginine and proline metabolism, and glycine, serine, and threonine metabolism were increased in the microbial genome of blueberry-fed mice. The down-regulated pathways correlated with the reduced *Firmicutes’* and increased *Bacteroidetes’* abundance in blueberry-fed groups. The enzymatic biotransformation of blueberries may also be relevant for xenobiotic metabolism, which may allow the conversion of many classes of compounds, including flavonoids, isoflavonoids, lignans, phenolic acids, fiber, and tannins [65,66]. In our study, we noticed an increase in meta-genomic functional pathways associated with xenobiotic degradation and benzoate degradation in mice fed a blueberry diet relative to those fed a control diet. Lee et al. reported the increased short-chain fatty acid (SCFA) production in blueberry-fed rats [58]. Changes in predicted pathways upon blueberry supplementation, especially the metabolism-related pathways, might change the production of SCFAs in these mice. 

## 5. Conclusions

In conclusion, our study demonstrated that blueberry supplementation in male and female mice led to compositional changes in the gut microbiota at all taxonomic levels. These changes also led to an alteration in the predicted metabolic pathways, which revealed stronger associations between specific pathway-enriched bacterial genomes and select microbes only in male mice. Thus, our study provides further support that the biological effects of blueberries are associated with alterations in the gut microbiota. The current study provides novel insights into the repertoire of these changes and advances our understanding of the effects of blueberry-enriched diets. The study also highlights diet-sex interactions in the gut microbiota. However, the present findings primarily describe the observed associations between changes in the microbiome and the predicted metabolic pathways, without any metabolic functional assessment, and are associative in nature. Future studies are needed to make causal inferences about the role of the altered microbiome and to ascertain the functional significance. These studies would ideally involve microbiome manipulation (via antibiotics) or transplantation (into germ-free animals). Future experiments involving human subjects that explore these relationships will be required to understand which findings are recapitulated in the context of dietary blueberry consumption. 

## Figures and Tables

**Table 1:** The diet compositions, showing the weight (g/kg), % by weight, and % by kcal of the dietary components.

Ingredients	7% Corn Oil Diet	5% Blueberry Diet
	TD.95092	TD.10679
	g/kg	g/kg
Casein	200.0	198.2
L-Cysteine	3	3
Corn Starch	397.5	351.9
Maltodextrin	132	132
Sucrose	100	100
Corn Oil	70.0	69.5
Cellulose	50.0	47.9
Min Mix (AIN-93G-MX(94046))	35	35
Vitamin Mix (AIN-93-VX(94047))	10	10
Choline Bitartrate	2.5	2.5
TBHQ, Anti-oxident	0.014	0.014
Blueberry Powder	-	50
**Both Diets**		
Macronutrient Info	% by weight	% kcal from
Protein	17.7	18.8
Carbohydrate	60.1	63.9
Fat	7.2	17.2
Kcal/g	3.8	3.8

**Table 2:** α-diversity indices at the phylum and genus levels (Data are expressed as mean ± SE). Statistical differences between the groups (F-BB (female mice fed a blueberry diet) (*n* = 9), F-Con (female mice fed a control diet) (*n* = 7), M-BB (male mice fed a blueberry diet) (*n* = 9), and M-Con (male mice fed a control diet) (*n* = 10) were determined by Two-Way ANOVA for sex and diet and their interaction (bold typed numbers state the significantly different indices).

**Phylum**							
**Index**	**F_BB**	**F_Con**	**M_BB**	**M_Con**	**Sex**	**Diet**	**Diet:Sex**
Chao1	5.444 ± 0.176	5.714 ± 0.184	6 ± 0	5.5 ± 0.167	0.225	0.381	**0.017**
Fisher	0.48 ± 0.017	0.5 ± 0.018	0.534 ± 0.001	0.485 ± 0.016	0.163	0.299	**0.025**
InvSimpson	2.22 ± 0.041	2.214 ± 0.099	2.176 ± 0.03	2.181 ± 0.067	0.526	0.955	0.924
Observed	5.444 ± 0.176	5.714 ± 0.184	6 ± 0	5.5 ± 0.167	0.225	0.381	**0.017**
Shannon	0.908 ± 0.025	0.929 ± 0.034	0.887 ± 0.022	0.915 ± 0.027	0.512	0.389	0.880
Simpson	0.548 ± 0.008	0.543 ± 0.022	0.54 ± 0.006	0.538 ± 0.015	0.599	0.737	0.890
**Genus**							
**Index**	**F_BB**	**F_Con**	**M_BB**	**M_Con**	**Sex**	**Diet**	**Diet:Sex**
Chao1	31.833 ± 0.677	34.286 ± 0.778	33.667 ± 1.003	34.833 ± 0.564	0.125	**0.022**	0.412
Fisher	3.368 ± 0.071	3.614 ± 0.091	3.568 ± 0.101	3.719 ± 0.065	0.071	**0.017**	0.570
InvSimpson	5.186 ± 0.233	5.49 ± 0.32	4.883 ± 0.21	6.277 ± 0.258	0.398	**0.001**	**0.041**
Observed	31.667 ± 0.601	34.143 ± 0.738	33.333 ± 0.882	34.6 ± 0.521	0.128	**0.009**	0.392
Shannon	2.091 ± 0.039	2.067 ± 0.061	2.124 ± 0.039	2.227 ± 0.028	**0.029**	0.191	0.128
Simpson	0.804 ± 0.009	0.814 ± 0.011	0.792 ± 0.009	0.838 ± 0.006	0.534	**0.001**	**0.046**

**Table 3:** The operational taxonomical unit (OTU) abundance at the genus level. F-BB (female mice fed a blueberry diet) (*n* = 9), F-Con (female mice fed a control diet) (*n* = 7), M-BB (male mice fed a blueberry diet) (*n* = 9), and M-Con (male mice fed a control diet) (*n* = 9). Data are expressed as mean ± SE. Statistical differences between the groups were determined by Two-Way ANOVA for sex, diet, and their interaction. Bold typed numbers indicate the significantly different indices.

Family_Genus	F_Con	F_BB	M_Con	M_BB	Sex	Diet	Diet × Sex
Clostridiales_Unassigned	12.93 ± 3.25	15.8 ± 1.04	12.42 ± 1.51	16.68 ± 1.85	0.954	0.065	0.720
Staphylococcaceae_Unassigned	0.04 ± 0.01	0.01 ± 0	0.02 ± 0.01	0 ± 0	0.064	**0.000**	0.434
Alcaligenaceae_Sutterella	0 ± 0	0.06 ± 0.04	0.02 ± 0.01	0.08 ± 0.04	0.652	**0.047**	0.985
Coriobacteriaceae_Adlercreutzia	0.29 ± 0.07	1.19 ± 0.15	0.38 ± 0.09	0.72 ± 0.15	**0.050**	**0.000**	**0.033**
Erysipelotrichaceae_Unassigned	0.07 ± 0.02	0.05 ± 0.01	0.05 ± 0.02	0.08 ± 0.02	0.740	0.615	0.131
Clostridiaceae_Unassigned	5.52 ± 1.59	8.35 ± 1.6	12.04 ± 1.3	6.06 ± 0.65	0.123	0.144	**0.002**
Staphylococcaceae_Staphylococcus	0.19 ± 0.04	0.04 ± 0.01	0.14 ± 0.04	0.02 ± 0.01	0.479	**0.000**	0.613
Lactobacillaceae_Lactobacillus	23.83 ± 3.73	9.93 ± 1.96	9.67 ± 2.6	4.47 ± 1.38	**0.001**	**0.001**	0.086
Ruminococcaceae_Oscillospira	1.8 ± 0.32	2.72 ± 0.39	2.39 ± 0.39	3.42 ± 0.29	0.132	**0.011**	0.885
Staphylococcaceae_Jeotgalicoccus	0.05 ± 0.01	0.01 ± 0.01	0.05 ± 0.01	0.02 ± 0.01	0.204	**0.002**	0.920
Dehalobacteriaceae_Dehalobacterium	0.07 ± 0.01	0.17 ± 0.03	0.12 ± 0.02	0.16 ± 0.04	0.589	**0.027**	0.311
X.Mogibacteriaceae._Unassigned	0.03 ± 0.01	0.11 ± 0.02	0.04 ± 0.01	0.1 ± 0.01	0.654	**0.000**	0.566
Ruminococcaceae_Ruminococcus	3.8 ± 1.24	1.12 ± 0.25	6.64 ± 1.26	2.46 ± 0.62	**0.018**	**0.001**	0.432
Lachnospiraceae_Dorea	0.05 ± 0.02	0.11 ± 0.02	0.02 ± 0.01	0.03 ± 0	**0.000**	**0.050**	0.064
Streptococcaceae_Lactococcus	0.95 ± 0.14	0.28 ± 0.04	0.86 ± 0.19	0.38 ± 0.14	0.695	**0.000**	0.503
Ruminococcaceae_Unassigned	2.84 ± 0.73	1.83 ± 0.21	3.28 ± 0.31	2.37 ± 0.36	0.162	**0.024**	0.901
Porphyromonadaceae_Parabacteroides	3.44 ± 0.72	6.44 ± 0.91	2.75 ± 0.45	3.74 ± 0.52	**0.007**	**0.008**	0.143
S24.7_Unassigned	27.48 ± 1.08	36.57 ± 1.32	30.15 ± 1.68	39.53 ± 1.23	0.167	**0.000**	0.920
Planococcaceae_Sporosarcina	0.01 ± 0	0 ± 0	0.01 ± 0	0.01 ± 0.01	**0.036**	0.647	0.458
Lachnospiraceae_Coprococcus	0.21 ± 0.03	0.92 ± 0.16	0.37 ± 0.08	1.44 ± 0.23	0.089	**0.000**	0.243
RF39_Unassigned	0.23 ± 0.15	0.57 ± 0.28	0.33 ± 0.08	1.11 ± 0.3	0.220	**0.014**	0.330
Christensenellaceae_Unassigned	0.46 ± 0.09	0.11 ± 0.03	0.28 ± 0.05	0.08 ± 0.01	0.114	**0.000**	0.123
Anaeroplasmataceae_Anaeroplasma	0.03 ± 0.02	0 ± 0	0.02 ± 0	0.01 ± 0	0.941	0.143	0.166
Lachnospiraceae_.Ruminococcus.	0.59 ± 0.14	0.35 ± 0.08	0.26 ± 0.04	0.72 ± 0.15	0.837	0.186	**0.002**
Lachnospiraceae_Unassigned	3.73 ± 1.24	4.94 ± 0.59	5.84 ± 0.74	5.42 ± 0.25	0.102	0.668	0.274
Turicibacteraceae_Turicibacter	0.18 ± 0.1	2.22 ± 0.91	1.51 ± 0.67	4.54 ± 0.76	**0.034**	**0.001**	0.508
Ruminococcaceae_Anaerotruncus	0.02 ± 0.01	0 ± 0	0.02 ± 0.01	0 ± 0	0.793	**0.000**	0.799
Deferribacteraceae_Mucispirillum	8.72 ± 1.5	3.75 ± 0.98	6.45 ± 1.02	2.71 ± 0.34	0.219	**0.000**	0.542
Corynebacteriaceae_Corynebacterium	0.55 ± 0.2	0.37 ± 0.24	0.73 ± 0.2	0.07 ± 0.04	0.879	**0.026**	0.211
Peptococcaceae_Unassigned	0.03 ± 0.01	0.06 ± 0.02	0.08 ± 0.02	0.08 ± 0.01	0.169	0.474	0.393
Peptococcaceae_rc4.4	0.76 ± 0.26	1.46 ± 0.3	0.83 ± 0.27	1.46 ± 0.39	0.941	**0.042**	0.908
Bacillales_Unassigned	0.03 ± 0.01	0 ± 0	0.01 ± 0	0.01 ± 0	0.351	**0.035**	**0.004**
Aerococcaceae_Facklamia	0.05 ± 0.01	0.04 ± 0.02	0.06 ± 0.01	0.01 ± 0.01	0.832	0.056	0.208
Moraxellaceae_Acinetobacter	0.02 ± 0.01	0 ± 0	0 ± 0	0.01 ± 0	0.258	0.200	**0.012**
Bacteroidaceae_Bacteroides	0.96 ± 0.36	0.33 ± 0.1	1.92 ± 0.52	1.95 ± 0.54	**0.005**	0.539	0.455
Peptostreptococcaceae_Unassigned	0.01 ± 0	0.01 ± 0	0.02 ± 0.01	0.01 ± 0.01	0.059	0.394	0.433
Clostridiaceae_Clostridium	0.07 ± 0.02	0.08 ± 0.02	0.21 ± 0.02	0.07 ± 0.01	**0.002**	**0.000**	**0.000**
